# Recognition of Affective Faces of Different Age Groups

**DOI:** 10.1093/geroni/igab046.2845

**Published:** 2021-12-17

**Authors:** Kim Jinhwi, Seunghee Park, Jongwan Kim

**Affiliations:** Jeonbuk National University, Jeonju, Cholla-bukto, Republic of Korea

## Abstract

It has been found that valence and arousal are the core affect dimensions in emotional structure. In this study, we hypothesized that there might be differences between different age groups in emotional structure using six facial expression stimuli (angry, disgusted, fearful, neutral, happy, and sad) of three age groups (young, middle-aged, and old). Unlike previous studies asking participants to rate subjective ratings or similarities between stimuli, participants in this study were required to determine whether stimulus pairs were the same or different emotions and reaction time and accuracy were measured for further analyses. We assumed that it would be harder when the stimulus pair is similar whereas it would be easier when the pair is different. The results showed that for the same emotion pair condition, the sad-sad pair had the lowest accuracy and the longest reaction time, while the happiness-happiness pair had the highest accuracy and the shortest reaction time. For the different emotion pairs, angry-disgusted and disgusted-sad was the lowest accuracy and the longest reaction time. For age of the stimuli effect, responses to the old faces had the lowest accuracy and the longest reaction time. The results suggest that identification of emotional stimuli might be affected by emotion category and age. Further study may need to recruit various age groups, because participants in the current study were mostly young adults.

